# Retention in care for type 2 diabetes management in Sub‐Saharan Africa: A systematic review

**DOI:** 10.1111/tmi.13859

**Published:** 2023-02-15

**Authors:** Anupam Garrib, Tsi Njim, Olukemi Adeyemi, Faith Moyo, Natalie Halloran, Huanyuan Luo, Duolao Wang, Joseph Okebe, Katie Bates, Victor Santana Santos, Kaushik Ramaiya, Shabbar Jaffar

**Affiliations:** ^1^ Department of Clinical Sciences Liverpool School of Tropical Medicine Liverpool UK; ^2^ Department of International Public Health Liverpool School of Tropical Medicine Liverpool UK; ^3^ Department of Medical Statistics, Informatics and Health Economics Medical University of Innsbruck Innsbruck Austria; ^4^ Department of Medicine Federal University of Sergipe Lagarto Brazil; ^5^ Health Science Graduate Program Federal University of Sergipe Aracaju Brazil; ^6^ Shree Hindu Mandal Hospital Dar es Salaam Tanzania; ^7^ UCL Institute for Global Health University College London London UK

**Keywords:** attrition, retention in care, retention rates, Sub‐Saharan Africa, systematic review, type 2 diabetes

## Abstract

**Objective:**

Diabetes prevalence has risen rapidly in Sub‐Saharan Africa, but rates of retention in diabetes care are poorly understood. We conducted a systematic review and meta‐analysis to determine rates of retention in care of persons with type 2 diabetes.

**Methods:**

We searched MEDLINE, Global Health and CINAHL online databases for cohort studies and randomised control trials (RCTs) published up to 12 October 2021, that reported retention in or attrition from care for patients with type 2 diabetes in Sub‐Saharan Africa. Retention was defined as persons diagnosed with diabetes who were alive and in care or with a known outcome, while attrition was defined as loss from care.

**Results:**

From 6559 articles identified, after title and abstract screening, 209 articles underwent full text review. Forty six papers met the inclusion criteria, comprising 22,610 participants. Twenty one articles were of RCTs of which 8 trials had 1 year or more of follow‐up and 25 articles were of non‐randomised studies of which 19 had 12 months or more of follow‐up. A total of 11 studies (5 RCTs and 6 non‐randomised) were assessed to be of good quality. Sixteen RCTs were done in secondary or tertiary care settings. Their pooled retention rate (95% CI) was 80% (77%, 84%) in the control arm. Four RCTs had been done in primary care settings and their pooled retention rate (95% CI) was 53% (45%, 62%) in the control arm. The setting of one trial was unclear. For non‐randomised studies, retention rates (95% CI) were 68% (62%, 75%) among 19 studies done in secondary and tertiary care settings, and 40% (33%, 49%) among the 6 studies done in primary care settings.

**Conclusion:**

Rates of retention in care of people living with diabetes are poor in primary care research settings.

## INTRODUCTION

About 1.6 million deaths worldwide are attributed to type 2 diabetes mellitus (hereon referred to as diabetes) annually [1, 2, 3, 4, 5, 6]. The prevalence of diabetes in Sub‐Saharan Africa (hereafter referred to as Africa) has risen rapidly [6, 7, 8], now standing between 3% and 10%, and the largest global increase in prevalence in the next two decades is expected to occur on this continent [3, 9]. Diabetes is affecting a younger and a poorer population in Africa than is the case in high‐income countries, with almost 80% of all diabetes‐related deaths occurring before people reach the age of 60 years, and high levels of morbidity from diabetes‐related complications [9, 10]. The effective control of diabetes is therefore a major challenge facing health services.

Prevention of morbidity and mortality from diabetes requires the disease to be controlled adequately. As a first step, this requires patients to be identified, linked to and retained in care. This is difficult in Africa, where health systems have typically focussed on acute management of infectious diseases and are ill‐prepared and ill‐resourced to respond to the chronic nature of care required for diabetes [11, 12, 13, 14]. The impact of this is seen in the low achievement of optimal diabetes treatment goals in the region [10]. For patients, the costs of transport and other related expenses can be catastrophic and accessing care regularly for diabetes presents a huge problem [15].

Experience with the HIV care programme suggests that provision of chronic care in Africa is challenging but achievable [16]. Unlike HIV, diabetes care does not have the dedicated resources HIV care has received. Unmet need for diabetes care is high with one cross‐sectional study finding that of all people with diabetes, only 40% had been diagnosed and 38% were on treatment [17]. With the rising burden of diabetes, knowledge of the rates of retention in care is essential for planning a response to this epidemic. We conducted this systematic review and meta‐analysis to assess the rates of retention in diabetes care for adults in Sub‐Saharan Africa.

## METHODS

The protocol for the review is registered on the PROSPERO database (registration number CRD42018112400). Institutional review board approval was not required since the information used was published and publicly available. We present our findings according to Preferred Reporting Items for Systematic Reviews and Meta‐Analysis (PRISMA) guidelines (Appendix 1).

### Search strategy and eligibility criteria

We searched MEDLINE, Global Health and CINAHL for articles published in English up to 12 October 2021 and reporting retention or attrition rates in patients with type‐2 diabetes managed in health facilities across Sub‐Saharan Africa. The main search terms were: ‘diabetes mellitus’, ‘dysglycaemia’, ‘hyperglycaemia’ and ‘hyperinsulinemia’ coupled with ‘retention in care’, ‘retention rates’, ‘attrition rates’, ‘lost to follow up’, ‘patient dropout’, ‘patient adherence’ and ‘patient compliance’. These were combined using Boolean characters ‘OR’ ‘AND’. We searched the reference lists of included papers for additional papers or cross‐referenced studies. A detailed description, including MESH terms, of the search is outlined in Appendix 2.

Two authors independently screened the titles and abstracts for relevance, and a third review author was consulted to resolve disagreements.

We included randomised trials, retrospective and prospective cohort studies. For studies with multiple publications, we selected the article that included outcomes relevant for the review. We excluded letters to the editor, reviews, editorials and commentaries, conference abstracts or unpublished studies, studies with fewer than 30 participants or less than 3 months of follow up, and studies or programme descriptions which followed up patients in diabetes care but did not have enough data to compute an estimate of the retention of patients in care or attrition rates. We included studies that reported on retention in care in type 2 diabetics where patients with hypertension and type 1 diabetes where also included and where possible, separated out the results in type 2 diabetics.

### Data extraction

Using pre‐tested data extraction forms, we independently extracted the following general information from each included article: surname of the first author; date of publication; study design; country of study; sample size; method of patient recruitment; type of intervention; age range of participants; sex; follow up period and the proportion of participants retained in care. Any differences between the two entries were resolved by discussion with a third author.

We contacted the corresponding authors of included articles where valuable information (retention rates or attrition rates for diabetes patients in care and sample size) was missing in the text or not in a retrievable format. Reminders were sent weekly and if there was no response after a month, the article was excluded.

### Definitions

We defined diabetes care as receiving any of the following interventions: drugs, advice or support, diet and lifestyle modification aimed at controlling blood sugar and reducing risk of complications in patients diagnosed with type 2 diabetes.

Retention was defined as being alive and in care at the end of the study or period of evaluation, or where the patient mortality was known and occurred in care. Retention was also considered as the converse of attrition, which is measured as a combination of loss from care or death. This loss from care is described where a participant has not returned or made any contact with the clinic for about 3–6 months. Rates of retention were derived from attrition by subtracting the reported attrition from 100%.

### Assessment of methodological quality and risk of bias

The quality of studies and the risk of bias were assessed independently by two authors using the quality assessment tool for observational studies and randomised control trials (RCTs) of the National Health Institute/National Heart, Lung, and Blood Institute. Any disagreements that occurred between the two reviewers in the course of assessing methodological quality and risk of bias were arbitrated by a third reviewer.

### Data synthesis and analysis

The primary analysis for all outcomes was by intention to‐treat where the denominator was the number of patients enrolled in the study. Participants who were lost to follow‐up were considered as not retained. We excluded multi‐site trials where we were not able to separate data for Sub‐Saharan African countries.

To calculate pooled retention rates by study arm at different follow‐up time points for RCTs, a generalised linear mixed model with a binomial distribution and log link function was used. The model had study arm (intervention arm or control arm), follow‐up time, interaction between study arm and follow‐up time as fixed effects, study as random effect. For cohort (non‐randomised) studies, the pooled retention rates at different follow‐up time points were also estimated using a similar generalised linear mixed model but only with follow‐up time as the sole predictor. Generalised linear mixed model was estimated using the Proc Glimmix in SAS version 9.4.

## RESULTS

**FIGURE 1:**
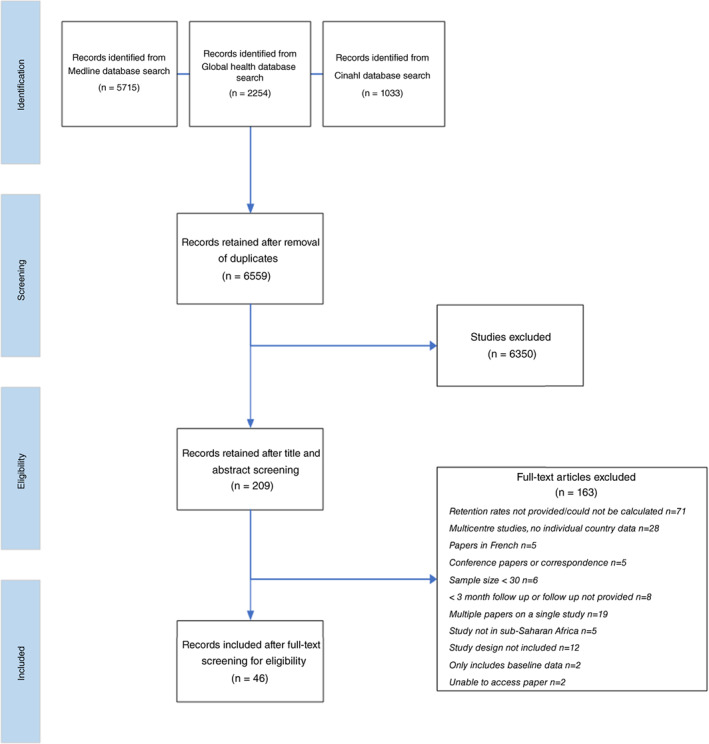
PRISMA flowchart

A total of 9002 articles were identified initially by the search (Figure 1 and Appendix 1). After removal of duplicates, 6559 studies remained. The titles and abstracts were screened to eliminate 6350 irrelevant articles. The full texts of the remaining 209 studies were scrutinised with 46 articles meeting the inclusion criteria. These comprised 21 RCTs and 25 cohort studies with total of 4557 and 18,053 evaluable participants, respectively. The studies had been conducted in 14 countries in Africa: Cameroon, Democratic Republic of Congo, Ethiopia, Ghana, Kenya, Mali, Malawi, Mauritius, Nigeria, Rwanda, South Africa, Sudan, Tanzania and Uganda.

Using the quality assessment tools for RCTs and observational studies of the National Health Institute/National Heart, Lung, and Blood Institute; five of the RCTs [18, 19, 20, 21, 22] were deemed to be of ‘good quality’ (score of 10–14 in quality assessment tool), 12 of the RCTs [23, 24, 25, 26, 27, 28, 29, 30, 31, 32, 33, 34] were ‘fair quality’ (score of 7–9 in quality assessment tool) and the remaining four studies [35, 36, 37, 38] were ‘low quality’. Six of the observational studies [39, 40, 41, 42, 43, 44] were of ‘good quality’ (score of 10–14), 15 [45, 46, 47, 48, 49, 50, 51, 52, 53, 54, 55, 56, 57, 58, 59] were of ‘fair quality’ (score of 7–9). The remaining five studies [60, 61, 62, 63, 64] were deemed ‘low quality’.

The characteristics of the RCTs included in the systematic review and meta‐analysis are given in Table 1 and the non‐randomised studies in Table 2. The range of follow‐up for patients retained in care varied from 3 to 24 months in the RCTs and 3 months to 14 years in the cohort studies. Retention in care as a converse measure of ‘attrition/loss to follow up’ was calculated from 18 studies [18, 30, 33, 34, 37, 41, 42, 43, 46, 48, 50, 51, 52, 55, 58, 60, 62, 65]. The results are reported by study design. One RCT [23] and five non‐randomised studies [40, 51, 55, 56, 58] reported retention in care as a primary outcome of the study.

**TABLE 1:** Summary of randomised controlled trials included in the review.

No.	Authors	Year	Country	Total sample size	Level of care	Age profiles (mean age (years) ± *SD*)	Sex profiles (% female)	Primary outcome of the trial	Type of intervention	Retention in care	
										Intervention arm	Control arm
1	Adibe et al [19]	2013	Nigeria	220	Tertiary	52.8 ± 8.2 in control 52.4 ± 7.6 in intervention	57.7%	Incremental cost and QALYs gained	Structured self‐care education and training versus usual care	At 6 months, 93% (102/110) At 12 months, 90% (99/110)	At 6 months, 89% (98/110) At 12 months, 85% (93/110)
2	Adjei et al [25]	2015	Ghana	200	Tertiary	Reported by age group	64.5%	BMI, systolic BP, diastolic BP, pulse rate, fasting glucose	Electronic reminders of appointments to patients, physician prompts of abnormal results.	At 6 months, 100% (100/100)	At 6 months, 88.0% (88/100)
3	Amendezo et al [26]	2017	Rwanda	251	Tertiary	50.9 ± 10.9	69.3%	HbA1c	Structured lifestyle educational training programme	At 12 months, 95% (117/123)	At 12 months, 88% (113/128)
4	Anyanwu et al [35]	2016	Nigeria	42	Tertiary	51.1 ± 1.9 in control 52.5 ± 2.2 in intervention	45.2%	Serum vitamin D and fasting plasma glucose	Vitamin D3 supplementation	At 3 months, 81% (17/21)	At 3 months, 76% (16/21)
5	Asante et al [31]	2020	Ghana	60	Tertiary	56.5 ± 9.8 in control 55.1 ± 10.9 in intervention	78.3%	Change in HbA1c	Mobile phone calls by a diabetes specialist nurse	At 3 months, 100% (30/30)	At 3 months, 100% (30/30)
6	Babiker et al [27]	2017	Sudan	97	Tertiary	50 ± 9 years (of *n =* 91)	80.2% (of *n =* 91)	Mean percent change of fasting plasma glucose and HbA1c	Gum Arabic dietary fibre supplementation	Overall retention at 3 months, 94% (91/97)	
7	David et al [22]	2021	Nigeria	108	Tertiary	51.8 ± 11.8	68.5%	HbA1c	Pharmacist led education and adherence support	At 6 months, 100% (54/54)	At 6 months, 100% (54/54)
8	Debussche et al [18]	2018	Mali	151	Secondary	51.1 ± 9.6 in control 53.9 ± 9.8 intervention	76.2%	HbA1c	Peer‐led structured patient education	At 6 months, 95% (72/76) At 12 months, 92% (70/76)	At 6 months, 96% (72/75) At 12 months, 93% (70/75)
9	Erku et al [38]	2017	Ethiopia	127	Tertiary	59.8 ± 13.5 in control 61.3 ± 11.4 in intervention	Unclear	Medication adherence	Pharmacist‐led medication therapy management	At 6 months, 87% (54/62)	At 6 months, 82% (53/65)
10	Essien et al [20]	2017	Nigeria	118	Tertiary	52.7 ± 10.5	60.2%	HbA1c	Diabetes self ‐management educational	At 6 months, 90% (53/59)	At 6 months, 86% (51/59) in control
11	Gathu et al [28]	2018	Kenya	140	Tertiary	48.8 ± 9.8	44.3%	HbA1c	Diabetes self ‐management educational	At 6 months, 79% (55/70)	At 6 months, 59% (41/70) in control
12	Hailu et al [36]	2018	Ethiopia	220	Tertiary	54 ± 10 in control 55 ± 10 in intervention	32.7%	HbA1c	Diabetes self ‐management educational	At 9 months, 67% (78/116)	At 9 months, 62% (64/104)
13	Idowu et al. [37]	2020	Nigeria	58	Tertiary	48.3 ± 9.4	65%	Pain intensity	Graded activity ± daily monitored walking	At 3 months, 86% (25/29)	At 3 months, 90% (26/29)
14	Labhardt et al [23]	2011	Cameroon	221 Of which 34 diabetics	Primary	Not reported separately for diabetes cohort	Not reported separately for diabetes cohort	Retention rates (defined as a least 12 visits within 12 months)	Patient contract Financial incentivisation Reminder letters	In all study participants: At 12 months, 60% in intervention 1 (financial incentive), 65% in intervention 2 (invitation letter), 29% in control In diabetics: overall retention at 12 months 44% (15/34)	
15	Mash et al [24]	2011	South Africa	1570	Community and Primary	56.1 ± 11.6	73.8%	Weight and HbA1c	Group diabetes education	At 12 months, 55% (391/710)	At 12 months 55% (475/860)
16	Muchiri et al. [32]	2021	South Africa	77	Tertiary	57.6 ± 6.8 in intervention 56.9 ± 6.4 in control	78%	HbA1c	Diabetes nutrition education programme	At 12 months, 62% (24/39)	At 12 months, 63% (24/38)
17	Owolabi et al. [33]	2019	South Africa	216	Primary	60.6 ± 11.6	84.3%	Change in mean randome blood sugar	Daily educational text messages	At 6 months, 91% (98/108)	At 6 months, 70% (76/108)
18	Thuita et al [34]	2020	Kenya	153	Tertiary	57.0 ± 10.9 in NEP group 55.0 ± 12.3 in NE group 56.0 ± 11.97 in control group	59.5%	Metabolic syndrome prevalence	Nutrition education (NE) versus nutrition education with peer support (NEP) versus standard care	At 6 months NE 96% (49/51) NEP 94% (48/51)	At 6 months 90% (46/51)
19	Val Olmen et al. [29]	2017	DRC, Cambodia, Philippines	1471, 506 in DRC^a^	Primary and community	Reported for those completing the study and those lost to follow up 59 ± 10 (completed) 63 ± 11 (ltfu)	67% in DRC	HbA1c	Text message self ‐management support	Overall retention in DRC at 24 months, 38% (191/506) (data not available separately for intervention and control arms)	
20	Van Rooijen et al. [30]	2010	South Africa	51	Tertiary	54.1 ± 6.3 in control 53.2 ± 6.5 intervention	58.8%	HbA1c	Educational and physical activity intervention	At 12 months, 85% (23/27)	At 12 months, 83% (20/24)
21	Van Rooijen et al [21]	2004	South Africa	158	Not clear	54 in exercise group 55 in relaxation group	100%	HbA1c	Exercise and relaxation interventions	At 12 weeks, 94% (75/80)	At 12 weeks, 96% (74/77)

^a^ Includes type 1 and type 2 diabetics.

**TABLE 2:** Summary of cohort (non‐randomised) studies included in the review.

No.	Study	Year	Country	Sample size	Level of care	Age profiles (mean age [years] ± *SD*)	Sex profiles (% female)	Primary outcome	Type of interventions	Retention in care
*Intervention studies*										
1	Adeniyi et al [47]	2010	Nigeria	77	Tertiary	Not reported for whole cohort	Not reported for whole cohort	Neuromusculoskeletal status	Therapeutic exercise programme	At 24 weeks, 56% (43/77)
2	Adeniyi et al [39]	2009	Nigeria	152	Tertiary	48 ± 9.6 years	Not clear	Sociodemographic determinants of attrition	Exercise programme	At 3 months in those recruited 45% (69/152) At 3 months in those that commenced the programme, 74% (69/93)^a^
3	Gill et al [46]	2008	South Africa	284	Primary	56 ± 11.0 years	80.0%	Glycaemic status, measured by HbA1c	Patient education and implementation of diabetes treatment protocol	At 18 months, 74% (210/284)
4	Ipingbemi et al [62]	2021	Nigeria	227	Tertiary	62.9 ± 11.6	80%	Treatment adherence	Pharmacist‐led educational intervention in those with HbA1c ≥7% versus usual care in those with HbA1c < 7%	At 6 months, 88% (106/121) in intervention group At 6 months, 90% (95/106) in control group
5	Labhardt et al [51]	2010	Cameroon	796 of which 144 had type 2 diabetes	Primary	60 ± 12 years	69%	Number of newly detected patients and retention of patients	Clinical staff training on management of diabetes and hypertension, provision of equipment and some drugs, and supervision	At 1 year 18.1% (63/349)^b^ At 2 years, 29% (234/796) (above includes both diabetes and hypertension patient)
6	Mshelia et al [63]	2007	Nigeria	220	Tertiary	51.4 ± 11.3 years for intervention arm; 50.8 ± 10.3 years for control arm	39.9% female (of those who completed the study 71/178)	Measures of morbidity, for example, obesity, overweight, hypertension	Participation in group health education, received longer consultation times	At 5 years, 81% (178/220)
7	Musicha et al [40]	2016	Malawi	2480 of which 339 were diabetic	Primary	Not reported for those who took up care	63.7%	Uptake of care and retention in care	Community based screening. Uptake of clinical care following screening.	Among diabetics (339): Retention at 3 months 45.1% Retention at 6 months 34.2% Retention at 12 months 27.% Retention at 18 months 10.9% Retention at 21 months 4.1%
8	Pastakia et al [41]	2017	Kenya	108 (89 htn, 10 htn and dm, 2 dm, 7 known dm/htn)	Primary and community	Not reported separately for diabetes care	42.0% female	Linkage frequency (percentage of patients who return for care after screening positive for either hypertension and/or diabetes)	Contextualised care delivery model, including peer support, education, treatment and economic sustainability	At 9 months, hypertension group 67%(60/89), hypertension and diabetes group 70% (7/10), diabetes group 100% (2/2) Overall retention at 9 months 70%(76/108) In patients with diabetes 75% (9/12)
9	Wroe et al [56]	2020	Malawi	2990 with NCDs of which 149 had diabetes^c^	Primary and community	For diabetes: 53 ± 17	64.4%	Retention outcome: 1 year survival is defined as patients known to be alive and retained in care 12 months after enrolment date.	Care delivered in an integrated chronic care clinic delivering care to patients with HIV and/or non‐communicable diseases	At 1 year, 78% (117/149) alive and in care Overall retention at 1 year 81% (121/149)
*Observational studies*										
10	Adua et al [45]	2017	Ghana	241	Tertiary	57.8 ± 10.9 years	58.9%	Glycaemic status	Measuring risk factors and response to treatment in type 2 diabetics	At 6 months, 66% (160/241)
11	Agboola‐Abu et al [48]	1999	Nigeria	60	Tertiary	Not reported for whole cohort	Not reported for whole cohort	Measures of diabetes control (fasting blood glucose, mean glycated haemoglobin)	Effects of medication on lipid profile and glycaemic control in new type 2 diabetics	At 24 weeks, 60% (36/60)
12	Wambui Charity et al [54]	2016	Kenya	164^d^	Tertiary	Median 33 (IQR 21–55)	59%	Adherence to self‐monitoring of blood glucose and HbA1c	Review of patients in a blood glucose self‐monitoring programme	At 12 months, 74% (121/164)
13	Ducorps et al [60]	1997	Cameroon	550 of which 408 had NIDDM (type 2 diabetes)^e^	Tertiary	46.9 ± 12.5 in whole cohort 48.9 ± 10.8 in NIDDM	38%	Classification of diabetes	Classification and investigation of different types of diabetes in Africa	At 43 months, 54% (295/550) Not reported separately for NIDDM(type 2 diabetes)
14	Elbagir et al [49]	2004	Sudan	86 Of which 59 were type 2 diabetics at diagnosis	Tertiary	33.9 ± 7.2 years (insulin treatment) 38.0 ± 5.5 years (diet/oral hypoglycaemic treatment)	53.5%	Serum fasting C peptide and HbA1c	Value of islet cell antibodies (ICA) and serum fasting C peptide investigation at diagnosis	At 24 months, 90% (77/86) At 24 months in type 2 diabetics 90% (53/59)
15	Katz et al [57]	2009	South Africa	257 with type 2 diabetes	Primary and secondary	Not reported	69%	Functional and clinical outcomes Appropriate specialist referrals Analysis of nurses knowledge	Training of primary care nurses, decision support, escalated scaling up of medication, enhanced specialist referral	At 2 years 77% (198/257)
16	Keeton et al [50]	2004	South Africa	62	Tertiary	Not clear	Not clear	Renal failure as a cause of mortality	Clinical evaluations and biochemical investigations	At 12 years, 95% (59/62)^f^ Mortality 80% (47/59)
17	Lester et al [52]	1993	Ethiopia	1386	Tertiary	Not clear	52% female	Clinical features, complications and prognosis of diabetes	Cross sectional survey, and retrospective review of records	At 14 years, 35% lost to follow up and 12% died Overall retention at 14 years 65%
18	McLarty et al [42]	1990	Tanzania	1250	Primary, secondary and tertiary	Not clear	29.8% female	5‐year survival rate	Prospective observational study	At 6 years, 77.9% (974/1250) Overall retention at 6 years is between 63% (including all who had not been seen in 6 months) and 87% (those who had not been seen in 3 years)
19	Pinchevsky et al [59]	2016	South Africa	666	Tertiary	64 ± 10.6 years (*n =* 261)	55.% female	HbA1c, blood pressure, low‐density lipoprotein (LDL)	Review of patient records	Overall retention at 48 months, 39% (261/666)
20	Sarfo‐Kantanka et al [43]	2018	Ghana	8330	Tertiary	54.2 ± 11.9 years (*n =* 7383)	63.8% female	Incidence and predictors of diabetic foot	No intervention	Overall retention at 12 months 89% (7383/8330)
21	Sobry et al [53]	2014	Kenya	Hypertension (1279) diabetes (100) and hypertension + diabetes (80)	Tertiary	Not clear	71.0% female overall 62% of those with diabetes	Blood pressure and glycaemic control	Descriptive study using prospectively collected routine data	Overall retention at 24 months, 68% (1006/1465) Not reported separately for diabetes
22	Tapela et al. [58]	2016	Rwanda	544	Primary	Not provided	56.1% female	Death, loss to follow‐up and HbA1c at intervals of 6, 12, 18 and 24 months.	Retrospective electronic medical record review	Retention at 6 months 93% Retention at 12 months 89% Retention at 18 months 88% Retention at 24 months 83%
23	Tapp et al [44]	2006	Mauritius	1126	Secondary	50 ± 11.0 years in responders, 54 ± 12.0 years in non‐responders	58.0% female in responders, 55.0% female in non‐responders	Prevalence of diabetic retinopathy	Follow up survey following complication screening	At 6 years, 47% (528/1126)
24	Tino et al [55]	2019	Uganda	1818	Secondary	Median age (IQR) (years) Retained 60 [30, 36, 50, 51, 52, 53, 54, 55, 56, 57, 58, 59, 60, 61, 62, 63, 64, 65, 66] Lost to follow up 53 ([44]; [45]; [46]; [47]; [48]; [49]; [50]; [51]; [52]; [53]; [54]; [55]; [56]; [57]; [58]; [59]; [60]; [61]; [62]; [63]; [64])	59% female (1066/1818)	Loss to follow up from diabetes care after initiation of treatment	Retrospective record review to determine loss to follow up of patients with type 2 diabetes in care	At 12 months after registration, 48% (873/1818) Over 13 years, 7% (128/1818) (defined as those who had not attended within 6 months of last appointment) Ltfu rate 34.9/1000 person years (95% CI: 33.2–36.6)
25	Viswanathan et al [61]	2010	Tanzania	155	Tertiary	Mean (*SD*) 55.6 ± 11.6 years	33.5% females (52/155)	Post amputation outcome and associated complications	Surgical intervention (amputation)	At 30 months, 69.0% (107/155)

^a^ A total of 152 patients were consecutively enrolled in the study. A total of 93 patients commenced the exercise programme, of which 69 completed the 12‐week exercise programme.

^b^ Including all those patients who had been recruited at least 15 months before assessment; lost to follow up defined as no consultation for >3 months.

^c^ Possibly includes some type 1 diabetes as 13% of patients with and NCD <15 years and 18% on insulin.

^d^ Does not differentiate between type 1 and type 2 diabetics, however only 6% were on oral medication.

^e^ Non‐insulin dependent diabetes mellitus (NIDDM) = type 2 diabetes.

^f^ While only 3 patients were lost to follow up, 47 patients were known to have died.

### RCTs testing novel diabetes management strategies against comparison groups among people living with diabetes

All of the RCTs enrolled persons with diabetes with the aim to test the effectiveness of novel intervention strategies for diabetes management: patient education and support programmes [18, 19, 20, 22, 24, 26, 28, 31, 32, 33, 34, 36, 38], medications [35, 65], facility‐based interventions including financial incentives [23], exercise programmes [21, 30, 37] and electronic reminders of appointments and self‐management [25, 29]. These approaches were designed to enhance adherence to anti‐diabetes medications, improve anthropometric and clinical outcomes (body mass index, glycaemia and HbA1C levels) and to retain people in care. The comparison group was typically standard care. Only one RCT reported a financial incentive partially covering costs of drugs [23] and in the remainder, provision of drugs was left to the health service.

Thirteen trials had a duration of follow‐up of less than 12 months and in 8 follow‐up was for 1 year or more. Of the 21 trials, 16 had been conducted in secondary or tertiary care [18, 19, 20, 22, 25, 26, 28, 30, 31, 32, 34, 35, 36, 37, 38, 65], comprising 2073 participants in total. Their pooled retention rate (95% CI) was 84% (81%, 88%) in the intervention arm and 80% (77%, 84%) in the control arm. Among these 16 trials, 5 trials comprising 750 participants had reported retention rates at 12 months of follow‐up [18, 19, 26, 30, 32]. Their pooled retention rates (95% CI) were 89% (85%, 93%) in the intervention arm and 85% (81%, 90%) in the control arm.

Four of 21 trials had been done in primary care settings [23, 24, 29, 33]. They comprised 2326 participants in total. Their pooled retention rate (95% CI) was 58% (51%, 66%) in the intervention arm and 53% (45%, 62%) in the control arm. In one of the 21 trials [21] the setting was unclear.

Of the 8 trials with more than 12 months follow‐up, 5 were done in secondary and tertiary care settings, with rates of retention ranging from 84% to 93% (Table 3) [18, 19, 26, 30, 32] and 3 had taken place in a primary care setting and reported retention rates of 44% [23] and 55% [24] at 12 months and 38% [29] at 2 years.

**TABLE 3:** Retention rates (%) by time of follow up for cohort (non‐randomised) studies.

		*n*	Months of follow‐up							
			3	6	9	12	18	24	4 years	>5 years
*Intervention studies*										
1	Adeniyi (2010) [47]	77		56						
2	Adeniyi (2009) [39]	152	45							
3	Gill (2008) [46]	284					74			
4	Ipingbemi (2021) [62]	227			89					
5	Labhardt (2010) [51]	144/796						29		
6	Mshelia (2007)	220								81
7	Musicha (2016) [40]	339/2480	45	34		27	11	4 (21 months)		
8	Pastakia (2017) [41]	12/108			75 (in t2d)					
9	Wroe (2020) [56]	149				78				
*Observational studies*										
10	Adua (2017) [45]	241		66						
11	Agboola (1999) [48]	60		60						
12	Charity (2016) [54]	164				74				
13	Ducorps (1997) [60]	408/550							54	
14	Elbagir (2004) [49]	59/86						90		
15	Katz (2009) [57]	257						77		
16	Keeton (2004) [50]*	62								95 (80% mortality)
17	Lester (1993) [52]	1386								65 (12% mortality)
18	McLarty (1990) [42]	1250								63
19	Pinchevsky (2016) [59]	666							39	
20	Sarfo‐Kantanka (2018) [43]	8330				89				
21	Sobry (2014) [53]	1465						68		
22	Tapela (2016) [58]	544		93		89	88	83		
23	Tapp (2006) [44]	1126								47
24	Tino (2019) [55]	1818				48				
25	Viswanathan (2010) [61]*	155/526								97 (31% mortality)
*Average retention*			45	62	70	70	58	69	47	

### Non‐randomised studies reporting rates of retention in care

There were 25 cohort studies. A total of 19 enrolled participants from or treated participants in secondary or tertiary care health facilities and 6 were conducted in primary care settings.

**FIGURE 2:**
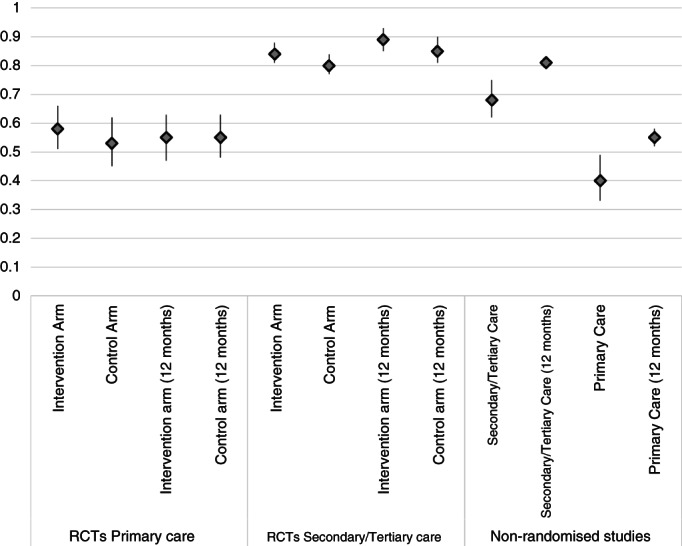
Graph of pooled retention rates

The 19 studies conducted in secondary and tertiary care facilities had varying durations of follow‐up. Five studies had follow‐up durations of 3–6 months and they reported retention ranging from 45% to 89% [39, 45, 47, 48, 62]. Three studies had 12 months of follow up and reported retention rates ranging from 48% [55] to 74% [54] and 89% [43]. Four studies had 2–3 years of follow‐up and reported retention rates between 68% and 90%. [49, 53, 57, 61] Three studies had 4–5 years of follow‐up and reported retention rates of 39% [59], 54% [60] and 81% [63]. Retention rates at 6 years of follow‐up in two studies that included both type 1 and type 2 diabetics were 47% [44] and 63% [42]. One study in South Africa had a 12‐year follow‐up and reported a high retention rate of 95% coupled however with a high mortality rate of almost 80% [50]. Finally, one study had a follow‐up period of 14 years [52]. It comprised both people living with type 1 and type 2 diabetes and had a retention rate of 65% and a mortality rate of 12%. The 19 studies had a combined sample size of 18,123 participants and the combined pooled retention rate was 68% (95% CI 62%, 75%) (Figure 2).

Of these 19 non‐randomised studies in tertiary/secondary care settings, just 3 reported retention at 12 months [43, 54, 55]. The pooled estimate (95% CI) was 81% (95% CI 80%, 82%).

There were six studies that took place in a primary care setting comprising 1568 participants. Their combined pooled retention rate was 40% (95% CI 33%, 49%). Two studies comprising 488 participants reported retention at 12 months [40, 56]. The pooled rate (95% CI) was 55% (52%, 58%).

## DISCUSSION

This systematic review shows that retention in research studies on diabetes care is poor in primary care settings in Sub‐Saharan Africa. The review involved studies done in research settings, where patients will have had good access to care, education about diabetes and medicines adherence support. Four of the primary care studies were randomised trials and even among these, the retention rate was low. If retention is a challenge in research settings, then good retention rates will be impossible to achieve in real‐life settings where access to services is limited: particularly for rural populations, shortages of drugs and diagnostics are common and patients have high out‐of‐pocket costs. Indeed, one author commented ‘it was quite tough and expensive to have sustained the participants through the study’ in reference to a RCT with 6 months of follow‐up [22].

Only about 5%–10% of people living with diabetes are estimated to be in care and given the limited routine glycaemia testing, almost all are identified after complications, that is, these patients will have had undiagnosed disease for some time [1]. Hence it is possible that patients who come into care (and were part of the studies in our systematic review) were relatively more motivated and that the low rates of retention we identified are over‐estimates. In contrast, about 80% of people living with HIV now know their HIV status. The majority are identified when they do not have symptoms and start treatment promptly [66]. Over 80% of those who are HIV positive are in care and virally suppressed. This large inequity in care between different chronic conditions raises major ethical dilemmas [67].

Our study also showed that retention in care was much better for people treated in tertiary and secondary care centres, particularly in randomised trials, reaching the levels seen in HIV care. The reasons for this higher retention are not clear. It is possible that these patients are experiencing complications and are symptomatic and therefore more likely to attend health services. It is also possible that these are a select group of patients or that there is a higher quality of care delivered at these settings. Health systems in these countries do not have the infrastructure to deliver chronic disease management at the primary care level, where the vast majority of people with diabetes will be treated [68]. From the experience with HIV and with diabetes in tertiary and secondary care, high retention is achievable, and primary care in low‐resource settings needs urgent strengthening to deliver this.

Achieving high retention in diabetes care is a fundamental first step to effective control. This is evident from the gains in HIV control that have been possible because of the remarkable improvements in retention in HIV care over the 20 years [69]. We do not know why retention in diabetes care is so low. Suggested factors include lack of availability of trained healthcare workers, costs of access to care, interruptions in drug supplies and poor provision of health education and counselling [17]. In settings where there are high out of pocket payments, patients may attend intermittently or may move between private and public providers. However our evidence included randomised trials which provide comparatively good quality care, including covering some of the costs of care. In a large study conducted in Malawi, people with diabetes were identified after systematic population‐based testing for diabetes. Those diagnosed were given free access to care at their local health centres, including free medicines and a dedicated clinician in a setting where medicines for diabetes were otherwise unavailable. Despite this, retention in care was less than 30% after 12 months [40]. Research is needed to better understand the reasons for poor retention, and approaches and interventions that could achieve good retention in a range of populations attending primary care. It is likely that education, support and empowerment focussed on patients and communities will be essential to increase understanding of the effects of diabetes, and to support patients to remain in care, as was done for HIV control.

The low retention in care we identified for diabetes is likely masking mortality due to diabetes, and it is essential that as well as interventions to improve retention in care, there is an urgent need to enhance medical recording systems at primary care level.

Limitations of this analysis include that we have used retention in research studies to estimate retention in diabetes care. Conducting research, particularly RCTs, at the primary care level in this context is difficult. As a result there was only a small number of studies we could use to estimate retention in primary care.

In research studies the care delivered is more resourced, staff will be trained and emphasis is put on retaining people in the study, reflecting what might be achieved in a routine setting with more resources and likely overestimating true retention in care. The definitions of retention in care did differ from study to study, and we also used loss to follow up/attrition to indicate retention, the definitions of which will also have varied by study. Retention in care is influenced by a number of factors including a lack of availability of services, drugs and well‐trained health care workers, accessibility of services and associated costs, and patient understanding of their condition; and these may differ from place to place. Our premise is that retaining people in care is the minimum and first requirement to achieve control of a condition. In summary, our systematic review shows low level of retention of care in studies of people living with diabetes and attending primary care services. We showed this in a number of settings, including in settings where high‐quality care was provided, such as randomised trials. Research is needed to understand reasons for poor retention and to develop interventions to improve retention in diabetes care, as retention is an essential first step to improve health outcomes of people with diabetes.

## FUNDING INFORMATION

This research was funded by the National Institute for Health Research (NIHR) (project reference 16/137/87) using UK aid from the UK Government to support global health research. The views expressed in this publication are those of the author(s) and not necessarily those of the NIHR or the UK Department of Health and Social Care. Katie Bates has been supported by a FWF Austrian Science Fund Lise Meitner Award [M‐3069‐B].

## Supporting information


**Data S1.** Appendices.
