# Supplementary material for: Retention in care for type 2 diabetes management in Sub‐Saharan Africa: A systematic review
Source: Trop Med Int Health. 2023 Feb 15;28(4):248–61. doi: 10.1111/tmi.13859 (PMC10947597; doi:10.1111/tmi.13859)
Supplement: Supplementary file 1 — Data S1. Appendices. [file TMI-28-248-s001.zip › TMI_13859_Appendix 3_Quality grading of RCTs.docx]

**Appendix 3: Quality Assessment Tool for Randomised control trials of the National Health Institute; Good quality studies (n=5)**

| **Criteria** | **Adibe** | **Debussche** | **Essien** | **Van Rooijen 2004** | **David** |
| --- | --- | --- | --- | --- | --- |
| Was the study described as randomized, a randomized trial, a randomized clinical trial, or an RCT? | Yes | Yes | Yes | Yes | Yes |
| Was the method of randomization adequate (i.e., use of randomly generated assignment)? | Yes | Yes | Yes | Yes | Yes |
| Was the treatment allocation concealed (so that assignments could not be predicted)? | No | Yes | Yes | Yes | Yes |
| Were study participants and providers blinded to treatment group assignment? | No | No | No | No | Yes |
| Were the people assessing the outcomes blinded to the participants' group assignments? | No | No | Yes | Yes | Not clear |
| Were the groups similar at baseline on important characteristics that could affect outcomes (e.g., demographics, risk factors, co-morbid conditions)? | Yes | Yes | Yes | Yes | Yes |
| Was the overall drop-out rate from the study at endpoint 20% or lower of the number allocated to treatment? | Yes | Yes | Yes | Yes | Yes |
| Was the differential drop-out rate (between treatment groups) at endpoint 15 percentage points or lower? | Yes | Yes | Yes | Yes | No |
| Was there high adherence to the intervention protocols for each treatment group? | Not clear | Yes | Yes | No | Yes |
| Were other interventions avoided or similar in the groups (e.g., similar background treatments)? | Yes | Yes | Yes | Not clear | Yes |
| Were outcomes assessed using valid and reliable measures, implemented consistently across all study participants? | Yes | Yes | Yes | Yes | Yes |
| Did the authors report that the sample size was sufficiently large to be able to detect a difference in the main outcome between groups with at least 80% power? | Yes | Yes | Yes | Yes | Yes |
| Were outcomes reported or subgroups analyzed prespecified (i.e., identified before analyses were conducted)? | Not clear | Yes | Yes | Yes | Yes |
| Were all randomized participants analyzed in the group to which they were originally assigned, i.e., did they use an intention-to-treat analysis? | Yes | Yes | Yes | No | Yes |
|  |  |  |  |  |  |
| Quality rating | Good | Good | Good | Good | Good |

**Fair studies (n=12)**

| **Adjei** | **Amendezo** | **Asante** | **Babiker** | **Gathu** | **Labhardt**  **2011** | **Muchiri** | **Owolabi** | **Thuita** | **Van Olmen** | **Van Rooijen 2010** | **Mash** |
| --- | --- | --- | --- | --- | --- | --- | --- | --- | --- | --- | --- |
| Yes | Yes | Yes | Yes | Yes | Yes | Yes | Yes | Yes | Yes | Yes | Yes |
| Yes | Not clear | yes | Not clear | Yes | Yes | Yes | Not clear | Yes | Yes | Yes | Yes |
| Not clear | No | Not clear | Yes | Yes | Yes | Yes | Yes | Yes | Yes | Yes | Not clear |
| No | No | No | Yes | No | No | Yes | No | Not stated | No | Not clear | No |
| Not clear | Yes | Not clear | Not clear | Yes | No | Yes | No | No | Not clear | Yes | No |
| Yes | Yes | Yes | Yes | Yes | Yes | Yes | Yes | Yes | Yes | No | No |
| Yes | Yes | Yes | Yes | No | No | No | Yes | Yes | No | Yes | No |
| Yes | Yes | Yes | Yes | No | No | Yes | No | Yes | No | Yes | no |
| Yes | Yes | Yes | Not clear | Not clear | Not clear | No | Yes | Not clear | Not clear | Not clear | No |
| Not clear | Not clear | Not clear | Yes | Not clear | Yes | Yes | Yes | Yes | Not clear | Not clear | Not clear |
| Yes | Yes | Yes | Yes | Yes | Yes | Yes | Yes | Yes | Not clear | Not clear | Yes |
| No | Yes | Yes | No | No | Yes | Yes | Yes | Yes | Yes | No | Yes |
| Yes | Not clear | Yes | Yes | Yes | Yes | Yes | Yes | Not clear | Yes | Yes | Yes |
| Yes | No | Yes | No | Yes | Yes | Yes | Yes | Yes | No | No | Yes |
|  |  |  |  |  |  |  |  |  |  |  |  |
| Fair | Fair | Fair | Fair | Fair | Fair | Fair | Fair | Fair | Fair | Fair | Fair |

**Poor studies (n=4)**

| **Anyanwu** | **Erku** | **Hailu** | **Idowu** |
| --- | --- | --- | --- |
| Yes | Yes | No | Yes |
| Not clear | Not clear | Yes | Not clear |
| Not clear | Not clear | Not clear | Not clear |
| No | Not clear | No | No |
| No | Not clear | Yes | No |
| Yes | Yes | Yes | Yes |
| Yes | Yes | No | Yes |
| Yes | Yes | No | Yes |
| No | Yes | No | Yes |
| Yes | Not clear | Not clear | Yes |
| Yes | Yes | Yes | Yes |
| Yes | No | No | Yes |
| Not clear | yes | Yes | Yes |
| Yes | Yes | Yes | Yes |
|  |  |  |  |
| Poor | Poor | Poor | Poor |
