# Supplementary material for: Retention in care for type 2 diabetes management in Sub‐Saharan Africa: A systematic review
Source: Trop Med Int Health. 2023 Feb 15;28(4):248–61. doi: 10.1111/tmi.13859 (PMC10947597; doi:10.1111/tmi.13859)
Supplement: Supplementary file 1 — Data S1. Appendices. [file TMI-28-248-s001.zip › TMI_13859_Appendix 4_Quality grading of Observational studies.docx]

**Appendix 4: Quality Assessment Tool for Observational, Cohort and Cross-Sectional Studies of the National Health Institute; Good articles (n=6)**

|  | **Criteria** |  | **Adeniyi 2009** | **McLarty** | **Musicha et al** | **Pastkia et al** | **Sarfo-Kantanka** | **Tapp** |
| --- | --- | --- | --- | --- | --- | --- | --- | --- |
| 1 | Was the research question or objective in this paper clearly stated? |  | Yes | Yes | Yes | Yes | Yes | Yes |
| 2 | Was the study population clearly specified and defined? |  | Yes | Yes | Yes | Yes | Yes | Yes |
| 3 | Was the participation rate of eligible persons at least 50%? |  | Yes | Not clear | Yes | Not clear | Yes | Yes |
| 4 | Were all the subjects selected or recruited from the same or similar populations (including the same time period)? Were inclusion and exclusion criteria for being in the study prespecified and applied uniformly to all participants? |  | Yes | Not clear | Yes | Yes | Yes | Yes |
| 5 | Was a sample size justification, power description, or variance and effect estimates provided? |  | No | No | No | No | No | No |
| 6 | For the analyses in this paper, were the exposure(s) of interest measured prior to the outcome(s) being measured? |  | Yes | Yes | Yes | Yes | Yes | Yes |
| 7 | Was the timeframe sufficient so that one could reasonably expect to see an association between exposure and outcome if it existed? |  | Yes | Yes | Yes | Yes | Yes | Yes |
| 8 | For exposures that can vary in amount or level, did the study examine different levels of the exposure as related to the outcome (e.g., categories of exposure, or exposure measured as continuous variable)? |  | Yes | Yes | Yes | Yes | Yes | Yes |
| 9 | Were the exposure measures (independent variables) clearly defined, valid, reliable, and implemented consistently across all study participants? |  | Yes | Yes | Yes | Yes | Yes | Yes |
| 10 | Was the exposure(s) assessed more than once over time? |  | No | Yes | No | Yes | Yes | Yes |
| 11 | Were the outcome measures (dependent variables) clearly defined, valid, reliable, and implemented consistently across all study participants? |  | Yes | Yes | Yes | Yes | Yes | Yes |
| 12 | Were the outcome assessors blinded to the exposure status of participants? |  | Not clear | No | Not clear | Not clear | No | Not applicable |
| 13 | Was loss to follow-up after baseline 20% or less? |  | No | No | No | Yes | Yes | No |
| 14 | Were key potential confounding variables measured and adjusted statistically for their impact on the relationship between exposure(s) and outcome(s)? |  | Yes | Yes | Yes | Not clear | Yes | Yes |
|  | Quality rating |  |  |  |  |  |  |  |
|  | Rating (Good, Fair or Poor) |  | Good | Good | Good | Good | Good | Good |

**Fair articles (n=15)**

| **Adeniyi 2010** | **Adua et al** | **Agboola-Abu** | **Wambui Charity** | **Elbagir** | **Gill** | **Katz** | **Keeton** | **Labhardt** | **Lester** | **Pinchevsky** | **Sobry** | **Tapela** | **Tino** | **Wroe** |  |
| --- | --- | --- | --- | --- | --- | --- | --- | --- | --- | --- | --- | --- | --- | --- | --- |
| Yes | Yes | Yes | Yes | Yes | Yes | Yes | Yes | Yes | Yes | Yes | Yes | Yes | Yes | Yes |  |
| Yes | Yes | Yes | Yes | Yes | No | Yes | Yes | No | Yes | Yes | Yes | Yes | Yes | Yes |  |
| Not clear | Not clear | Not clear | Not clear | Yes | Not clear | Not clear | Not clear | Not clear | Not clear | yes | Not clear | No | Yes | Yes |  |
| Yes | Yes | Yes | Yes | Yes | Yes | Yes | Yes | Yes | Yes | Yes | Yes | yes | Yes | Yes |  |
|  |  |  |  |  |  |  |  |  |  |  |  |  |  |  |  |
| No | No | No | No | No | No | No | No | No | No | No | No | No | No | No |  |
| Yes | Yes | Yes | Yes | Yes | Yes | Yes | Yes | Yes | Yes | Yes | No | Yes | Yes | Yes |  |
| Yes | Yes | Yes | Yes | Yes | Yes | Yes | Yes | Yes | Yes | Yes | Yes | Yes | Yes | Yes |  |
| Yes | Yes | Yes | Yes | Yes | Yes | Yes | Yes | Yes | Not applicable | No | Not applicable | No | Yes | No |  |
| Yes | Yes | Yes | Yes | Yes | Yes | Yes | Yes | Not clear | Yes | Not applicable | Not applicable | Yes | Yes | Yes |  |
| Yes | No | Yes | Yes | Yes | Yes | Yes | Yes | No | Yes | No | No | Not applicable | Yes | No |  |
| Yes | Yes | Yes | Yes | Yes | Yes | Yes | Yes | Yes | Yes | Yes | Yes | Yes | Yes | Yes |  |
| No | Not clear | Not clear | No | No | Not clear | No | Not applicable | No | Not clear | No | No | No | Not applicable | No |  |
| No | No | Yes | No | Yes | No | No | No | No | No | No | No | Yes | No | No |  |
| No | Yes | No | Not clear | No | No | No | No | Yes | No | Yes | No | No | Yes | No |  |
|  |  |  |  |  |  |  |  |  |  |  |  |  |  |  |  |
| Fair | Fair | Fair | Fair | Fair | Fair | Fair | Fair | Fair | Fair | Fair | Fair | Fair | Fair | Fair |  |

**Poor articles (n=5)**

| **Ducorps** | **Ipingbemi** | **Mshelia** | **Nwamaka Onyechi** | **Viswanathan** |  |
| --- | --- | --- | --- | --- | --- |
| Yes | Yes | Yes | Yes | Yes |  |
| No | Yes | Yes | Yes | Yes |  |
| Not clear | Yes | Not clear | Not clear | Not clear |  |
| Yes | Yes | Yes | Yes | Yes |  |
|  |  |  |  |  |  |
| No | Yes | No | No | No |  |
| Not clear | Yes | Yes | Yes | Yes |  |
| Yes | Yes | Yes | Yes | Yes |  |
| Not clear | No | No | Not applicable | Not applicable |  |
| Yes | Yes | Yes | Yes | Not applicable |  |
| Yes | No | No | No | Not applicable |  |
| Yes | Yes | No | Yes | Yes |  |
| Not clear | Not applicable | No | Not clear | No |  |
| No | Yes | Yes | Yes | Yes |  |
| No | No | No | No | No |  |
|  |  |  |  |  |  |
| Poor | Poor | Poor | Poor | Poor |  |
