# Supplementary material for: Retention in care for type 2 diabetes management in Sub‐Saharan Africa: A systematic review
Source: Trop Med Int Health. 2023 Feb 15;28(4):248–61. doi: 10.1111/tmi.13859 (PMC10947597; doi:10.1111/tmi.13859)
Supplement: Supplementary file 1 — Data S1. Appendices. [file TMI-28-248-s001.zip › TMI_13859_Appendix 2_Search_Strategy_corrected.docx]

**Appendix 2: Search strategies**

***Electronic sources***

Online databases: Medline, Global Health and CINAHL were searched using the search strategies in Table 1 and 2 from the date of creation of the database to 03/06/2019 and updated from June 2019 to October 2021 (12/10/2021), for relevant abstracts of articles.

**Search strategy for Medline database**

| **Searches** | **Search combinations** | **Search terms** | **Number of hits (search 1)** | **Number of hits (search 2)** |
| --- | --- | --- | --- | --- |
| S1 |  | “MH Diabetes mellitus+” OR “MH Diabetes mellitus, Type 2+” OR “MH Diabetes mellitus, Type 1+” | 400,749 | 39,451 |
| S2 |  | "Diabetes mellitus" OR "diabetes mellitus type 2" OR diabet* OR diabetes OR "type 2 diabetes" OR “type 1 diabetes” OR “type 1 diabetes mellitus” OR "type 2 diabetes mellitus" OR diabetic OR "type 2 diabetic" OR “type 1 diabetic” OR dysglycemia OR dysglycaemia OR hyperglycemia OR hyperglycaemia OR glucose OR "insulin resistance" OR insulin OR hyperinsulinemia OR hyperinsulinaemia | 1,183,531 | 172,375 |
| S3 | S1 OR S2 |  | 1,184,841 | 172,519 |
| S4 |  | MH Retention in care | 19 | 165 |
| S5 |  | MH "Patient Compliance+" OR MH "Treatment Adherence and Compliance+" | 226,358 | 23,005 |
| S6 |  | "patient compliance" OR "treatment adherence and compliance" OR “adherence" OR "Retention in care" OR "retention rate*" OR Retention OR "attrition rate*" OR "lost to follow up” OR “lost to follow-up” OR “loss to follow up” OR “lost to follow-up” OR attrition OR "loss from care" OR dropout OR "patient dropout*" OR adherence OR compliance OR refusal OR "non adherence" OR “non-adherence” OR "non compliance" OR “non-compliance” OR “follow up” OR “follow-up” OR “followed up” OR “followed-up” OR “longitudinal” OR “prospective” OR “cohort” OR “trial” OR “randomised trial” OR “randomised control trial” OR “mortality” OR “survival” | 4,377,427 | 808,377 |
| S7 | S4 OR S5 OR S6 |  | 4,466,549 | 817,471 |
| S8 |  | “MH Africa South of the Sahara+" OR “MH Africa, Western+” OR “MH South Africa+” OR “MH Africa, Eastern+” OR “MH Africa, Southern+” OR “MH Africa, Central+” | 193,573 | 26,530 |
| S9 |  | “Africa south of the sahara” OR Africa OR Angola OR Benin OR Botswana OR Burkina Faso OR Burundi OR Cameroon OR Canary Islands OR Cape Verde OR Central African Republic OR Chad OR Comoros OR Congo OR Democratic Republic of Congo OR Equatorial Guinea OR Eritrea OR Ethiopia OR Gabon OR Gambia OR Ghana OR Guinea OR Guinea Bissau OR Ivory Coast OR Cote d’Ivoire OR Kenya OR Lesotho OR Liberia OR Libya OR Madagascar OR Malawi OR Mali OR Mauritania OR Mauritius OR Mayotte OR Mozambique OR Mozambique OR Namibia OR Niger OR Nigeria OR Principe OR Reunion OR Rwanda OR Sao Tome OR Senegal OR Seychelles OR Sierra Leone OR South Africa OR St Helena OR Swaziland OR Tanzania OR Togo OR Uganda OR Western Sahara OR Zaire OR Zambia OR Zimbabwe OR Central Africa OR Central African OR West Africa OR West African OR Western Africa OR Western African OR East Africa OR East African OR Eastern Africa OR Eastern African OR South African OR Southern Africa OR Southern African OR subSaharan Africa OR subSaharan African OR subSaharan Africa OR subSaharan African | 576,431 | 98,591 |
| S10 | S8 OR S9 |  | 576,541 | 98,997 |
| S11 | S3 AND S7 AND S10 |  | 4,057 | 1658 |

**Search strategy for Global Health database**

| **Searches** | **Search combinations** | **Search terms** | **Number of hits** |  |
| --- | --- | --- | --- | --- |
| S1 |  | "Diabetes mellitus" OR "diabetes mellitus type 2" OR diabetes OR diabet* OR "type 2 diabetes" OR “type 1 diabetes” OR “type 1 diabetes mellitus” OR "type 2 diabetes mellitus" OR diabetic OR "type 2 diabetic" OR “type 1 diabetic” OR dysglycemia OR dysglycaemia OR hyperglycemia OR hyperglycaemia OR glucose OR "insulin resistance" OR insulin OR hyperinsulinemia OR hyperinsulinaemia | 209,190 | 25,781 |
| S2 |  | "patient compliance" OR "treatment adherence and compliance" OR “adherence" OR "Retention in care" OR "retention rate*" OR Retention OR "attrition rate*" OR "lost to follow up” OR “lost to follow-up” OR “loss to follow up” OR “lost to follow-up” OR attrition OR "loss from care" OR dropout OR "patient dropout*" OR adherence OR compliance OR refusal OR "non adherence" OR “non-adherence” OR "non compliance" OR “non-compliance” OR “follow up” OR “follow-up” OR “followed up” OR “followed-up” OR “longitudinal” OR “prospective” OR “cohort” OR “trial” OR “randomised trial” OR “randomised control trial” OR “mortality” OR “survival” | 546,239 | 89,257 |
| S3 |  | “Africa south of the sahara” OR Africa OR Angola OR Benin OR Botswana OR Burkina Faso OR Burundi OR Cameroon OR Canary Islands OR Cape Verde OR Central African Republic OR Chad OR Comoros OR Congo OR Democratic Republic of Congo OR Equatorial Guinea OR Eritrea OR Ethiopia OR Gabon OR Gambia OR Ghana OR Guinea OR Guinea Bissau OR Ivory Coast OR Cote d’Ivoire OR Kenya OR Lesotho OR Liberia OR Libya OR Madagascar OR Malawi OR Mali OR Mauritania OR Mauritius OR Mayotte OR Morocco OR Mozambique OR Mozambique OR Namibia OR Niger OR Nigeria OR Principe OR Reunion OR Rwanda OR Sao Tome OR Senegal OR Seychelles OR Sierra Leone OR South Africa OR St Helena OR Swaziland OR Tanzania OR Togo OR Uganda OR Western Sahara OR Zaire OR Zambia OR Zimbabwe OR Central Africa OR Central African OR West Africa OR West African OR Western Africa OR Western African OR East Africa OR East African OR Eastern Africa OR Eastern African OR South African OR Southern Africa OR Southern African OR subSaharan Africa OR subSaharan African OR subSaharan Africa OR subSaharan African | 272,531 | 32,772 |
| S4 | S1 AND S2 AND S3 |  | 1,800 | 454 |

**Search strategy for CINAHL Complete**

| **Searches** | **Search combinations** | **Search terms** | **Number of hits** |  |
| --- | --- | --- | --- | --- |
| S1 |  | (MH "Diabetes Mellitus+") OR (MH "Diabetes Mellitus, Type 2") OR (MH "Diabetes Mellitus, Type 1+") | 141,976 | 19,148 |
| S2 |  | "Diabetes mellitus" OR "diabetes mellitus type 2" OR diabetes OR diabet* OR "type 2 diabetes" OR “type 1 diabetes” OR “type 1 diabetes mellitus” OR "type 2 diabetes mellitus" OR diabetic OR "type 2 diabetic" OR “type 1 diabetic” OR dysglycemia OR dysglycaemia OR hyperglycemia OR hyperglycaemia OR glucose OR "insulin resistance" OR insulin OR hyperinsulinemia OR hyperinsulinaemia | 248,223 | 48,859 |
| S3 | S1 OR S2 |  | 248,796 | 48,982 |
| S4 |  | (MH "Patient Compliance+") OR (MH "Medication Compliance") | 43,734 | 6,120 |
| S5 |  | "patient compliance" OR "treatment adherence and compliance" OR “adherence" OR "Retention in care" OR "retention rate*" OR Retention OR "attrition rate*" OR "lost to follow up” OR “lost to follow-up” OR “loss to follow up” OR “lost to follow-up” OR attrition OR "loss from care" OR dropout OR "patient dropout*" OR adherence OR compliance OR refusal OR "non adherence" OR “non-adherence” OR "non compliance" OR “non-compliance” OR “follow up” OR “follow-up” OR “followed up” OR “followed-up” OR “longitudinal” OR “prospective” OR “cohort” OR “trial” OR “randomised trial” OR “randomised control trial” OR “mortality” OR “survival” | 1,103,355 | 254,486 |
| S6 | S4 OR S5 |  | 1,103,355 | 254,486 |
| S7 |  | (MH "Africa South of the Sahara+") OR (MH "Africa, Southern+") OR (MH "Africa, Eastern+") OR (MH "Africa, Central+") OR (MH "Africa, Western+") OR (MH "South Africa") | 57,720 | 12,000 |
| S8 |  | “Africa south of the sahara” OR Africa OR Angola OR Benin OR Botswana OR Burkina Faso OR Burundi OR Cameroon OR Canary Islands OR Cape Verde OR Central African Republic OR Chad OR Comoros OR Congo OR Democratic Republic of Congo OR Equatorial Guinea OR Eritrea OR Ethiopia OR Gabon OR Gambia OR Ghana OR Guinea OR Guinea Bissau OR Ivory Coast OR Cote d’Ivoire OR Kenya OR Lesotho OR Liberia OR Libya OR Madagascar OR Malawi OR Mali OR Mauritania OR Mauritius OR Mayotte OR Morocco OR Mozambique OR Mozambique OR Namibia OR Niger OR Nigeria OR Principe OR Reunion OR Rwanda OR Sao Tome OR Senegal OR Seychelles OR Sierra Leone OR South Africa OR St Helena OR Swaziland OR Tanzania OR Togo OR Uganda OR Western Sahara OR Zaire OR Zambia OR Zimbabwe OR Central Africa OR Central African OR West Africa OR West African OR Western Africa OR Western African OR East Africa OR East African OR Eastern Africa OR Eastern African OR South African OR Southern Africa OR Southern African OR subSaharan Africa OR subSaharan African OR subSaharan Africa OR subSaharan African | 83,363 | 20,264 |
| S9 | S7 OR S8 |  | 84,886 | 20,462 |
| S10 | S3 AND S6 AND S9 |  | 706 | 327 |
